# Supplementary material for: Modeling glioblastoma heterogeneity as a dynamic network of cell states
Source: Mol Syst Biol. 2021 Sep 16;17(9):e10105. doi: 10.15252/msb.202010105 (PMC8444284; doi:10.15252/msb.202010105)
Supplement: Supplementary file 5 — Source Data for Figure 3 [file MSB-17-e10105-s001.zip › Figure3A_sourcedata/GSEA_3065/nosigdb_state4.GseaPreranked.1622718430994/IVY_CT.html]

Details for gene set IVY\_CT[GSEA]

|  || Dataset | state4 |
| Phenotype | NoPhenotypeAvailable |
| Upregulated in class | na\_neg |
| GeneSet | IVY\_CT |
| Enrichment Score (ES) | -0.7010698 |
| Normalized Enrichment Score (NES) | -2.6261358 |
| Nominal p-value | 0.0 |
| FDR q-value | 0.0 |
| FWER p-Value | 0.0 |
Table: GSEA Results Summary

  

Fig 1: Enrichment plot: IVY\_CT      
 Profile of the Running ES Score & Positions of GeneSet Members on the Rank Ordered List

  

| PROBE | GENE SYMBOL | GENE\_TITLE | RANK IN GENE LIST | RANK METRIC SCORE | RUNNING ES | CORE ENRICHMENT || 1 | ID3 |  |  | 2 | 0.730 | 0.0213 | No |
| 2 | METRN |  |  | 14 | 0.462 | 0.0332 | No |
| 3 | SOX9 |  |  | 48 | 0.299 | 0.0366 | No |
| 4 | TSC22D4 |  |  | 49 | 0.298 | 0.0454 | No |
| 5 | TTYH1 |  |  | 100 | 0.249 | 0.0446 | No |
| 6 | FAM181B |  |  | 130 | 0.232 | 0.0468 | No |
| 7 | GATM |  |  | 156 | 0.219 | 0.0492 | No |
| 8 | LRRN3 |  |  | 181 | 0.211 | 0.0515 | No |
| 9 | DCLK2 |  |  | 250 | 0.186 | 0.0459 | No |
| 10 | EDNRB |  |  | 271 | 0.180 | 0.0480 | No |
| 11 | SALL1 |  |  | 286 | 0.177 | 0.0509 | No |
| 12 | KLHDC8A |  |  | 300 | 0.174 | 0.0540 | No |
| 13 | PLP1 |  |  | 330 | 0.169 | 0.0542 | No |
| 14 | ABAT |  |  | 435 | 0.145 | 0.0416 | No |
| 15 | FIBIN |  |  | 568 | 0.129 | 0.0238 | No |
| 16 | MLC1 |  |  | 594 | 0.126 | 0.0235 | No |
| 17 | KCNIP1 |  |  | 603 | 0.125 | 0.0259 | No |
| 18 | WSCD1 |  |  | 605 | 0.125 | 0.0294 | No |
| 19 | C1orf61 |  |  | 803 | 0.104 | 0.0003 | No |
| 20 | BBOX1 |  |  | 823 | 0.102 | 0.0003 | No |
| 21 | SOX21 |  |  | 930 | 0.093 | -0.0143 | No |
| 22 | SOX2 |  |  | 975 | 0.090 | -0.0188 | No |
| 23 | CSPG5 |  |  | 1035 | 0.086 | -0.0259 | No |
| 24 | SEMA6A |  |  | 1073 | 0.083 | -0.0295 | No |
| 25 | PCBP4 |  |  | 1188 | 0.077 | -0.0458 | No |
| 26 | ETV1 |  |  | 1199 | 0.076 | -0.0452 | No |
| 27 | RAMP1 |  |  | 1283 | 0.072 | -0.0566 | No |
| 28 | SCD5 |  |  | 1290 | 0.071 | -0.0555 | No |
| 29 | NPAS3 |  |  | 1483 | 0.062 | -0.0850 | No |
| 30 | TCF12 |  |  | 1488 | 0.062 | -0.0838 | No |
| 31 | POU3F2 |  |  | 1531 | 0.061 | -0.0889 | No |
| 32 | NES |  |  | 1551 | 0.060 | -0.0902 | No |
| 33 | MSI1 |  |  | 1744 | 0.054 | -0.1199 | No |
| 34 | ITGB8 |  |  | 1765 | 0.053 | -0.1216 | No |
| 35 | CKB |  |  | 1774 | 0.053 | -0.1213 | No |
| 36 | DDR1 |  |  | 1941 | 0.048 | -0.1470 | No |
| 37 | KCNQ2 |  |  | 2023 | 0.046 | -0.1589 | No |
| 38 | NLGN3 |  |  | 2183 | 0.042 | -0.1836 | No |
| 39 | SDC3 |  |  | 2214 | 0.041 | -0.1873 | No |
| 40 | NLGN1 |  |  | 2316 | 0.039 | -0.2026 | No |
| 41 | ATP6V0E2 |  |  | 2393 | 0.037 | -0.2139 | No |
| 42 | RFX4 |  |  | 2454 | 0.036 | -0.2226 | No |
| 43 | MATN2 |  |  | 2545 | 0.034 | -0.2363 | No |
| 44 | MEX3A |  |  | 2759 | 0.030 | -0.2702 | No |
| 45 | CDKN2C |  |  | 2822 | 0.028 | -0.2795 | No |
| 46 | LPL |  |  | 3121 | 0.023 | -0.3275 | No |
| 47 | FREM2 |  |  | 3238 | 0.020 | -0.3458 | No |
| 48 | LFNG |  |  | 3245 | 0.020 | -0.3462 | No |
| 49 | GAS1 |  |  | 3553 | 0.011 | -0.3960 | No |
| 50 | OLIG1 |  |  | 3854 | -0.002 | -0.4449 | No |
| 51 | SOX8 |  |  | 3882 | -0.003 | -0.4492 | No |
| 52 | SPATA6 |  |  | 4039 | -0.012 | -0.4744 | No |
| 53 | NXPH1 |  |  | 4058 | -0.013 | -0.4769 | No |
| 54 | DBI |  |  | 4122 | -0.017 | -0.4867 | No |
| 55 | VCAN |  |  | 4158 | -0.019 | -0.4918 | No |
| 56 | CRISPLD1 |  |  | 4306 | -0.029 | -0.5150 | No |
| 57 | ID4 |  |  | 4329 | -0.031 | -0.5176 | No |
| 58 | BCHE |  |  | 4672 | -0.057 | -0.5718 | No |
| 59 | NRM |  |  | 4765 | -0.065 | -0.5849 | No |
| 60 | PCDHGC3 |  |  | 4939 | -0.078 | -0.6108 | No |
| 61 | SCRG1 |  |  | 4970 | -0.080 | -0.6133 | No |
| 62 | RNASEH2A |  |  | 5241 | -0.105 | -0.6543 | No |
| 63 | PTN |  |  | 5261 | -0.107 | -0.6542 | No |
| 64 | EGFR |  |  | 5316 | -0.110 | -0.6598 | No |
| 65 | METTL7B |  |  | 5354 | -0.114 | -0.6624 | No |
| 66 | HES6 |  |  | 5392 | -0.118 | -0.6650 | No |
| 67 | IGF2BP3 |  |  | 5436 | -0.122 | -0.6684 | No |
| 68 | FADS2 |  |  | 5544 | -0.135 | -0.6818 | No |
| 69 | CREB5 |  |  | 5611 | -0.146 | -0.6883 | No |
| 70 | CDCA8 |  |  | 5642 | -0.151 | -0.6887 | No |
| 71 | SRPX |  |  | 5662 | -0.154 | -0.6873 | No |
| 72 | TTK |  |  | 5684 | -0.157 | -0.6861 | No |
| 73 | IGFBP2 |  |  | 5777 | -0.179 | -0.6958 | Yes |
| 74 | NOVA1 |  |  | 5789 | -0.181 | -0.6922 | Yes |
| 75 | PTPRZ1 |  |  | 5811 | -0.186 | -0.6901 | Yes |
| 76 | CCNE2 |  |  | 5827 | -0.190 | -0.6870 | Yes |
| 77 | BLM |  |  | 5833 | -0.191 | -0.6821 | Yes |
| 78 | PRC1 |  |  | 5835 | -0.192 | -0.6766 | Yes |
| 79 | SAPCD2 |  |  | 5845 | -0.194 | -0.6723 | Yes |
| 80 | CDCA7 |  |  | 5863 | -0.198 | -0.6693 | Yes |
| 81 | SOX11 |  |  | 5885 | -0.203 | -0.6667 | Yes |
| 82 | BUB1B |  |  | 5901 | -0.207 | -0.6630 | Yes |
| 83 | FAM111B |  |  | 5918 | -0.211 | -0.6594 | Yes |
| 84 | DLL3 |  |  | 5963 | -0.226 | -0.6598 | Yes |
| 85 | DTL |  |  | 5975 | -0.229 | -0.6549 | Yes |
| 86 | KIF2C |  |  | 5996 | -0.237 | -0.6511 | Yes |
| 87 | KIF14 |  |  | 6006 | -0.240 | -0.6455 | Yes |
| 88 | AURKA |  |  | 6026 | -0.248 | -0.6412 | Yes |
| 89 | BUB1 |  |  | 6045 | -0.266 | -0.6363 | Yes |
| 90 | FBXO5 |  |  | 6084 | -0.287 | -0.6340 | Yes |
| 91 | NUF2 |  |  | 6093 | -0.293 | -0.6266 | Yes |
| 92 | CKAP2L |  |  | 6108 | -0.303 | -0.6199 | Yes |
| 93 | MCM10 |  |  | 6110 | -0.305 | -0.6111 | Yes |
| 94 | COL9A3 |  |  | 6111 | -0.305 | -0.6021 | Yes |
| 95 | MELK |  |  | 6117 | -0.312 | -0.5936 | Yes |
| 96 | DSEL |  |  | 6121 | -0.316 | -0.5848 | Yes |
| 97 | ASF1B |  |  | 6127 | -0.320 | -0.5761 | Yes |
| 98 | LMNB1 |  |  | 6134 | -0.325 | -0.5675 | Yes |
| 99 | CDCA2 |  |  | 6135 | -0.326 | -0.5578 | Yes |
| 100 | TACC3 |  |  | 6138 | -0.328 | -0.5484 | Yes |
| 101 | CCNB2 |  |  | 6140 | -0.329 | -0.5389 | Yes |
| 102 | NDC80 |  |  | 6142 | -0.330 | -0.5292 | Yes |
| 103 | CDC20 |  |  | 6154 | -0.344 | -0.5208 | Yes |
| 104 | DLGAP5 |  |  | 6156 | -0.345 | -0.5108 | Yes |
| 105 | KIF15 |  |  | 6158 | -0.346 | -0.5007 | Yes |
| 106 | EZH2 |  |  | 6159 | -0.348 | -0.4904 | Yes |
| 107 | KIF4A |  |  | 6164 | -0.355 | -0.4805 | Yes |
| 108 | CDCA3 |  |  | 6165 | -0.355 | -0.4700 | Yes |
| 109 | RAD51AP1 |  |  | 6167 | -0.357 | -0.4596 | Yes |
| 110 | CENPK |  |  | 6169 | -0.363 | -0.4490 | Yes |
| 111 | CENPM |  |  | 6179 | -0.386 | -0.4391 | Yes |
| 112 | DNMT1 |  |  | 6182 | -0.396 | -0.4277 | Yes |
| 113 | MYBL2 |  |  | 6190 | -0.408 | -0.4167 | Yes |
| 114 | GINS2 |  |  | 6194 | -0.420 | -0.4048 | Yes |
| 115 | UHRF1 |  |  | 6198 | -0.436 | -0.3924 | Yes |
| 116 | RRM2 |  |  | 6199 | -0.438 | -0.3794 | Yes |
| 117 | DHFR |  |  | 6200 | -0.441 | -0.3664 | Yes |
| 118 | ESCO2 |  |  | 6206 | -0.463 | -0.3535 | Yes |
| 119 | PBK |  |  | 6207 | -0.465 | -0.3397 | Yes |
| 120 | FANCI |  |  | 6209 | -0.469 | -0.3260 | Yes |
| 121 | BCAN |  |  | 6211 | -0.481 | -0.3119 | Yes |
| 122 | BIRC5 |  |  | 6214 | -0.484 | -0.2979 | Yes |
| 123 | GTSE1 |  |  | 6217 | -0.490 | -0.2837 | Yes |
| 124 | CLSPN |  |  | 6218 | -0.490 | -0.2692 | Yes |
| 125 | SMC4 |  |  | 6219 | -0.490 | -0.2547 | Yes |
| 126 | NUSAP1 |  |  | 6223 | -0.507 | -0.2402 | Yes |
| 127 | CENPE |  |  | 6226 | -0.511 | -0.2254 | Yes |
| 128 | NCAPG |  |  | 6227 | -0.513 | -0.2102 | Yes |
| 129 | HIST1H1D |  |  | 6241 | -0.601 | -0.1945 | Yes |
| 130 | PTTG1 |  |  | 6245 | -0.622 | -0.1766 | Yes |
| 131 | CDK1 |  |  | 6246 | -0.627 | -0.1580 | Yes |
| 132 | HIST1H1B |  |  | 6249 | -0.646 | -0.1392 | Yes |
| 133 | TPX2 |  |  | 6253 | -0.667 | -0.1200 | Yes |
| 134 | ASPM |  |  | 6254 | -0.680 | -0.0998 | Yes |
| 135 | UBE2C |  |  | 6255 | -0.750 | -0.0776 | Yes |
| 136 | HMGB2 |  |  | 6258 | -0.766 | -0.0553 | Yes |
| 137 | TOP2A |  |  | 6260 | -0.814 | -0.0313 | Yes |
| 138 | CENPF |  |  | 6263 | -1.068 | 0.0000 | Yes |
Table: GSEA details [plain text format]

  

Fig 2: IVY\_CT: Random ES distribution      
 Gene set null distribution of ES for **IVY\_CT**
